# Early Recognition and Management of Flecainide Toxicity: A Case Report and Literature Review

**DOI:** 10.7759/cureus.73683

**Published:** 2024-11-14

**Authors:** Abdulrahman K Alraee, Tarique S Chachar, Ammar S Shaaban, Husam A Noor

**Affiliations:** 1 Cardiology, Mohammed Bin Khalifa Bin Salman Al Khalifa Specialist Cardiac Centre, Awali, BHR; 2 Cardiothoracic Surgery, Mohammed Bin Khalifa Bin Salman Al Khalifa Specialist Cardiac Centre, Awali, BHR

**Keywords:** antiarrhythmic drugs, atrial fibrillation, cardiovascular toxicity, flecainide, flecainide toxicity, overdose, sodium channel blocker

## Abstract

Flecainide acetate is classified as a class IC antiarrhythmic medication according to the Vaughan-Williams classification, primarily used to manage both ventricular and supraventricular tachycardia. It is commonly employed for pharmacological cardioversion of atrial fibrillation (AF) and is frequently used in the “pill-in-the-pocket” approach for on-demand rhythm control. Despite its efficacy, flecainide is associated with significant adverse effects, including cardiac arrest, dysrhythmias, and heart failure. The presence of renal impairment or drug-drug interactions can exacerbate these side effects. Although rare, the risk of cardiogenic shock in flecainide toxicity is noteworthy. Given the potential for life-threatening hemodynamic compromise, often manifesting as ventricular arrhythmias like ventricular tachycardia or ventricular fibrillation, emergency physicians should maintain a high index of suspicion for flecainide toxicity in patients using the drug. Early recognition is crucial, as delayed diagnosis and treatment can be fatal. This report presents a case of an elderly female patient who presented with wide QRS complex tachycardia and hyponatremia. Her baseline rhythm was restored without the need for cardioversion, defibrillation, or pacing following the administration of sodium bicarbonate.

## Introduction

Flecainide, a lipophilic antiarrhythmic medication, is classified as a class IC agent and is effective in treating supraventricular tachycardia, atrial fibrillation (AF), and ventricular arrhythmias. It is widely used for the pharmacological cardioversion of AF and is commonly employed in the “pill-in-the-pocket” strategy for rapid, on-demand rhythm control [1]. Flecainide exerts its effects by slowing phase 0 in fast sodium channels in a rate-dependent manner, which leads to a reduction in the cardiac action potential upstroke. This impairs impulse conduction, particularly in the His-Purkinje system and ventricular myocardium [2].

Despite its clinical benefits, flecainide has a narrow therapeutic index (0.2-1.0 mcg/mL), which predisposes patients to toxicity. Toxicity can lead to wide-complex tachycardia, ventricular fibrillation (VF), AV nodal block, bradyarrhythmia, and asystole. Electrocardiographic findings in toxicity typically include a prolonged PR interval, broad QRS complex, and prolonged QTc intervals. Since serum drug levels can take days to weeks to report, clinical suspicion and ECG findings are crucial for diagnosing toxicity [3]. Mortality associated with severe flecainide toxicity is reported to be 22.5% [4].

Given the narrow therapeutic index of flecainide, patients on this medication must be closely monitored, particularly those with renal impairment, which increases the risk of toxicity. The primary treatment for flecainide overdose is the administration of sodium bicarbonate, alongside supportive care [5]. Other management strategies, such as intravenous lipid emulsion (ILE) and the use of mechanical circulatory support, are also discussed. We present a case to emphasize the importance for emergency practitioners to promptly diagnose and treat flecainide toxicity when patients receiving flecainide therapy present with wide-complex tachyarrhythmia.

## Case presentation

A 67-year-old female with a history of paroxysmal AF, hypertension, dyslipidemia, hypothyroidism, severe mitral and tricuspid regurgitation, and an implantable cardiac defibrillator (ICD) placed in 2019 following cardiac arrest from idiopathic ventricular arrhythmia, was referred to our tertiary care cardiac center with a two-day history of intermittent dizziness, epigastric pain, and nausea. Her medications included apixaban 5 mg orally twice daily, verapamil 240 mg orally once daily, flecainide 100 mg orally twice daily, bisoprolol 2.5 mg orally once daily, ramipril 2.5 mg orally once daily, torsemide 20 mg orally twice daily, spironolactone 50 mg orally once daily, rosuvastatin 20 mg orally once daily, metformin 500 mg orally once daily, and thyroxine 200 mcg orally once daily. She had also recently started self-medicating with milk of magnesia and other herbal laxatives for chronic constipation.

On examination, she was alert, oriented, and hemodynamically stable with an initial heart rate of 106 bpm, blood pressure of 96/71 mmHg, and normal oxygen saturation on room air. Auscultation revealed a pansystolic murmur over the mitral area radiating to the axilla, without lung congestion. Abdominal and neurological examinations were unremarkable. ICD interrogation showed no VF or VT tracing, and no shock was delivered.

**Figure 1:**
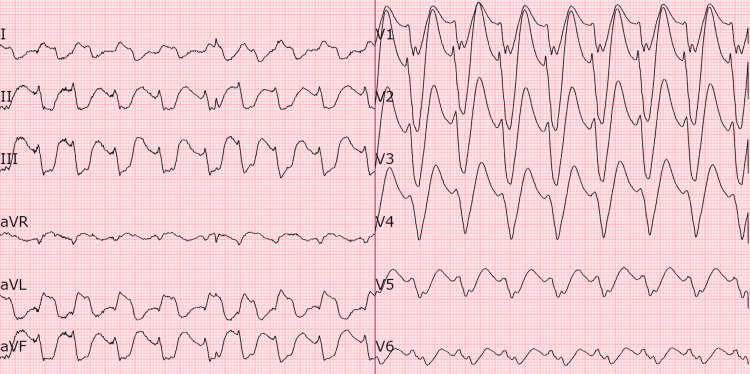
ECG on admission showing wide complex tachycardia with left axis deviation, LBBB morphology, and a markedly prolonged QRS duration (>200 ms) LBBB, left bundle branch block

On presentation, a 12-lead ECG (Figure 1) showed a wide complex rhythm with left axis deviation, left bundle branch block (LBBB) morphology, and a markedly prolonged QRS duration of >200 ms. Laboratory results revealed hyponatremia (sodium 128 mmol/L), hypomagnesemia (magnesium 0.67 mmol/L), normal potassium levels, normal renal function tests, normal thyroid function tests, and normal cardiac enzyme levels. Amylase and lipase levels were not checked. Transthoracic echocardiography demonstrated normal left ventricular (LV) systolic function, Grade I LV diastolic dysfunction, and severe mitral and tricuspid regurgitation, with a severely dilated left atrium. Chest X-ray revealed clear lung fields with no signs of significant pulmonary edema. A serum flecainide level drawn five days after presentation to the emergency department was found to be elevated at 2.05 mg/mL (reference range: 0.20-1.00 mg/ml; toxic threshold: >1.50 mg/ml), confirming flecainide toxicity.

**Figure 2:**
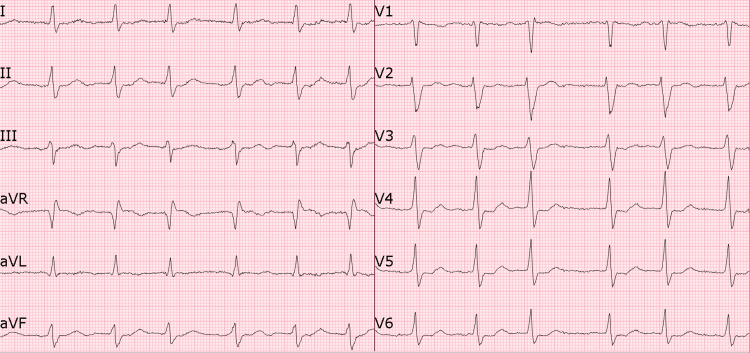
ECG post-treatment showing AF with a controlled heart rate, a narrower QRS complex (130 ms), and slow repolarization (QTc 497 ms) AF, atrial fibrillation

Given the patient’s use of flecainide 100 mg orally twice daily for idiopathic ventricular arrhythmia, ECG findings consistent with flecainide toxicity, and the presence of hyponatremia, flecainide toxicity was suspected. She was immediately treated with 100 mEq of 8.4% sodium bicarbonate, followed by an infusion of 0.45% sodium chloride with 75 mEq sodium bicarbonate at 75 ml/hr. The infusion was stopped after 18 hours when she became symptom-free, and her ECG showed her baseline AF with controlled heart rate, a narrower QRS complex of 130 ms, and slow repolarization (QTc 497 ms) (Figure 2).

**Figure 3:**
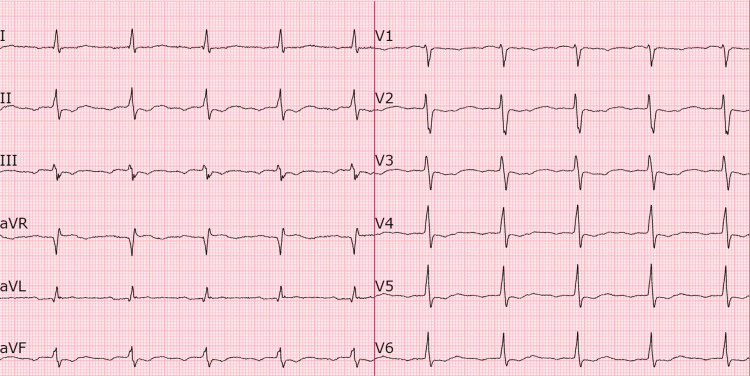
ECG on discharge showing resolution of all flecainide toxicity-associated morphology

The patient also received 4 g of magnesium sulfate to correct hypomagnesemia. Her electrolytes, as tested five days after presentation, revealed normalized levels (Na+ 136 mmol/L and Mg 0.87 mmol/L). A pre-discharge ECG showed complete resolution of the morphological abnormalities associated with flecainide toxicity (Figure 3). The patient reported full resolution of her presenting symptoms.

## Discussion

Flecainide acetate, a class IC antiarrhythmic medication according to the Vaughan-Williams classification, was initially developed as a novel anesthetic agent in 1972. It received FDA approval in 1985 for the treatment of sustained ventricular tachycardia (VT) [6]. Flecainide is considered a pro-arrhythmic agent, with the potential to induce pro-arrhythmic events, particularly in individuals with pre-existing cardiac conditions. As such, the FDA has issued a black box warning for flecainide, contraindicating its use in patients with structural heart disease. In the case of our patient, the presence of significant structural heart disease should have prompted consideration of switching to an alternative pharmacological agent.

Oral flecainide is rapidly absorbed, with plasma levels peaking within two to four hours. Its half-life ranges from seven to 23 hours in individuals with normal kidney function, but extends to 58 hours in end-stage renal failure due to reduced clearance. Flecainide is metabolized by CYP2D6 and CYP1A2, making drug interactions involving these pathways important to monitor. Approximately 86% of oral flecainide is excreted via the urine, highlighting the need for careful renal function monitoring and dose adjustments in patients with renal impairment [6]. In the case of our patient, her recent heavy use of laxatives for constipation may have contributed to an electrolyte imbalance, which could have predisposed her to flecainide toxicity. Additionally, the patient was using over-the-counter herbal supplements, which often contain unregulated ingredients and may lead to unexpected drug-drug interactions.

Flecainide exerts its effects through rate-dependent inhibition of phase 0 in fast sodium channels, with a strong affinity for open-state sodium channels. This action reduces the upstroke of the cardiac action potential, decreasing the conduction velocity of electrical impulses, particularly in the His-Purkinje system and ventricular myocardium. The anti-arrhythmic effectiveness of flecainide is closely linked to changes in QRS duration. The delayed unbinding kinetics from open-state Na+ channels during diastole and its high affinity for these channels explain the prolongation of refractoriness and slowed recovery time during cardiac diastole [1]. Additionally, flecainide blocks the opening of potassium channels, particularly the fast component of the K+ delayed rectifier current (IKr), extending the action potential duration (APD) in ventricular and atrial muscle fibers. However, in Purkinje fibers, flecainide reduces the APD due to sodium channel blockade [1]. Recent studies suggest that flecainide inhibits ryanodine receptor activation, thereby reducing the spontaneous release of Ca2+ from the sarcoplasmic reticulum, which can decrease afterdepolarization and triggered activity. This mechanism is thought to contribute to its therapeutic efficacy in treating catecholaminergic polymorphic VT [7].

The increased risk of toxicity associated with flecainide is due to its narrow therapeutic index (0.2-1.0 mcg/mL). Toxic symptoms can occur in some patients even at serum concentrations as low as 0.7 mcg/mL or higher [8]. Although class IC antiarrhythmic toxicity is extremely rare, accounting for only approximately 0.1% of all intoxications [9], the reported mortality rate is as high as 22.5% [4], emphasizing the critical need for early detection and treatment. Factors such as renal impairment and drug interactions involving CYP2D6 and CYP1A2 metabolic pathways are major contributors to flecainide toxicity. Electrolyte abnormalities and acidosis can also exacerbate or trigger toxicity. Additionally, hyponatremia may precipitate flecainide toxicity even at therapeutic doses [10]. Concomitant hypokalemia and hypomagnesemia further increase the risk of ventricular tachyarrhythmias. Toxicity manifestations include a variety of noncardiac symptoms, such as nausea, vomiting, and seizures, alongside cardiac symptoms like bradycardia, QRS widening, and ventricular tachyarrhythmia [4]. Wong and Wu reported flecainide-induced encephalopathy in a case of drug-drug interaction with psychiatric medications [11]. In our case, the patient presented with nausea, epigastric pain, and dizziness, which were likely extra-cardiac manifestations of flecainide toxicity, confirmed when the symptoms resolved completely with successful treatment.

Flecainide causes delayed conduction across the AV node, His-Purkinje system, and ventricles due to excessive sodium channel blockade. This can lead to serious arrhythmias, including asystole, VT, VF, and AV block. During supraventricular tachycardia, conduction slowing often results in a widened QRS complex with right or LBBB morphology, which complicates detection and increases the risk of misdiagnosis and inappropriate treatment [3]. The QRS duration on ECG is a crucial factor in evaluating the severity of flecainide toxicity. A QRS duration of ≤200 ms is typically associated with right bundle branch block, and these patients often experience rapid recovery without the need for mechanical circulatory support. In contrast, patients with LBBB morphology and a QRS duration >200 ms are more likely to require mechanical circulatory support and face an increased risk of mortality [12].

Addressing flecainide toxicity, particularly in severe cases marked by hemodynamic instability, requires a comprehensive and multidisciplinary approach. Key strategies include the administration of sodium bicarbonate, correction of electrolyte imbalances, use of ILE, minimizing patient agitation, providing mechanical circulatory support, and offering extracorporeal life support for critical patients who do not respond to pharmacological interventions.

Sodium bicarbonate is considered a first-line treatment for flecainide toxicity when the QRS duration exceeds 100 ms, typically administered in doses of 1-2 mEq/kg. It works by increasing extracellular sodium levels, which compete with flecainide for binding to sodium channels, and by raising serum pH, thereby enhancing the electrochemical gradient across cell membranes. This combination of sodium loading and alkalinization effectively reverses sodium channel blockade and narrows the QRS interval [13]. In our case, we initiated treatment with 100 mEq of 8.4% sodium bicarbonate, followed by a continuous infusion of 0.45% sodium chloride with 75 mEq of sodium bicarbonate at a rate of 75 mL/hr. The infusion was discontinued after 18 hours when the patient’s 12-lead ECG showed a return to a narrower QRS complex (QRS 130 ms) with slower repolarization (QTc 497 ms). Electrolyte imbalances were also corrected.

ILE therapy has emerged as a promising intervention for flecainide toxicity, particularly in severe cases where conventional treatments may prove insufficient. ILE works by creating a “lipid sink” that sequesters lipophilic drugs like flecainide, reducing their free concentration in plasma and mitigating their toxic effects on cardiac tissues. Additionally, ILE can serve as a direct energy source for the myocardium, which is particularly beneficial in cases of drug-induced cardiac depression [14]. The typical administration protocol includes an initial bolus followed by a continuous infusion, and its effectiveness in reversing cardiotoxicity is supported by several case reports and clinical observations. However, while ILE shows promise, the exact mechanism, optimal dosing, and long-term safety profile in the context of flecainide toxicity remain under investigation. Its use is generally recommended as part of a multimodal approach when conventional treatments fail [14].

Extracorporeal membrane oxygenation (ECMO) has been utilized as a form of mechanical circulatory support in severe cases of flecainide toxicity, particularly when conventional medical interventions fail. ECMO is preferred in these scenarios due to its ability to function independently of a stable cardiac rhythm. It ensures adequate perfusion of vital organs, including the liver and kidneys, which supports drug clearance and aids in the patient’s overall recovery [8]. A case report highlights the use of ECMO for 26 hours in a patient with an unintentional flecainide overdose, facilitating drug metabolism and removal while maintaining cardiac output and organ perfusion. After 12 days in intensive care, the patient was discharged without any long-term complications [9].

## Conclusions

This case underscores the critical role of emergency physicians in swiftly identifying and managing flecainide toxicity, given its potential to induce life-threatening arrhythmias. Suspecting flecainide toxicity is essential when patients on flecainide therapy present with nonspecific symptoms and a significantly widened QRS complex on ECG, particularly when accompanied by electrolyte abnormalities, renal impairment, or recent medication changes. Physicians must be equipped to initiate a multimodal treatment strategy, incorporating sodium bicarbonate, electrolyte correction, ILE, and mechanical circulatory support such as ECMO in severe cases. Early recognition and intervention are vital to preventing clinical deterioration and improving patient outcomes, highlighting the importance of heightened awareness and preparedness in emergency settings.
